# Editorial: Novel perspectives on the NLRP3 inflammasome

**DOI:** 10.3389/fimmu.2023.1211385

**Published:** 2023-05-12

**Authors:** Ying Liang, Kuo-Feng Hua

**Affiliations:** ^1^ Department of Toxicology and Cancer Biology, University of Kentucky, Lexington, KY, United States; ^2^ Department of Biotechnology and Animal Science, National Ilan University, Ilan, Taiwan; ^3^ Department of Medical Research, China Medical University Hospital, China Medical University, Taichung, Taiwan

**Keywords:** NLRP3 inflammasome, metabolic diseases, radiation therapy, Duchenne muscular dystrophy, diffuse large B-cell lymphoma, bibliometric study

The intracellular sensor NACHT, LRR, and PYD domain-containing protein 3 (NLRP3) inflammasome is a protein complex composed of NLRP3, apoptosis-associated speck-like protein containing a CARD (ASC) and cysteine protease caspase-1. The NLRP3 inflammasome is activated in response to infectious agents and sterile disease-relevant stimuli, which leads to the activation of caspase-1. Activated caspase-1 induces the cleavage of interleukin-1β (IL-1β) and IL-18 precursors to generate their mature forms (1). Caspase-1 and noncanonical inflammasome-activated caspase-11 induce cleavage of gasdermin D, which induces plasma membrane pore formation and leads to a lytic form of cell death called pyroptosis (Pan et al.).

Aberrant activation of the NLRP3 inflammasome induces inflammatory responses and promotes the development of a variety of diseases (2). For example, the NLRP3 inflammasome can be activated by sterile metabolic danger signals (e.g., cholesterol crystals, uric acid crystals and fatty acids) to induce metabolic diseases, including cardiovascular diseases, gout and diabetes. The NLRP3 inflammasome has a major influence on the development of heart failure, atherosclerosis and atrial fibrillation (3). One of the interesting topics in cardiovascular research is the study of the heart-brain interaction. The NLRP3 inflammasome can be activated by pressure overload and controls neural signals that may improve cardiovascular disease by modulating cardiac inflammation (4). Recent bibliometric studies indicate that NLRP3 inflammasome has been of great interest in the field of ischemic stroke and neurological disease (5, 6). Growing evidence highlights NLRP3 inflammasome is a promising therapeutic target in treating ischemic stroke and neurological disease. Investigations on the NLRP3 inflammasome in neurodegenerative disease have become increasingly popular recently, as it responds to misfolded or aggregated proteins that are commonly deposited in the brain, the hallmark of many neurodegenerative diseases (7). The microbiota plays important roles in the pathophysiology of many diseases. Recent studies have noted that the microbiota-gut-NLRP3 inflammasome-brain axis regulates brain homeostasis and the development of neurodegenerative diseases (Rutsch et al.).

A bibliometric study published in this Research Topic revealed the involvement of the NLRP3 inflammasome in acute respiratory distress syndrome, especially in COVID-19-associated acute respiratory distress syndrome, which is a novel topic that has shown rapidly increasing interest (Xiao et al.). The components of SARS-CoV-2 have been demonstrated to activate the NLRP3 inflammasome and induce excessive inflammatory responses. Notably, the activation level of the NLRP3 inflammasome is associated with COVID-19 severity in patients (8). Another interesting article published in this Research Topic characterized the relationship between the NLRP3 inflammasome and radiation-induced tissue injury and proposed prevention strategies targeting the NLRP3 inflammasome to improve radiation therapy in the treatment of various malignancies (Cheng et al.). Moreover, a new pathogenic role of the NLRP3 inflammasome in the muscle wasting disease Duchenne muscular dystrophy was reported. Inhibition of the NLRP3 inflammasome by MCC950 can significantly attenuate myonecrosis and fibrosis in mice with Duchenne muscular dystrophy disease, and MCC950-treated mice showed a more mature myofiber phenotype and exhibited enhanced force and resistance to fatigue (Dubuisson et al.). These findings indicate the possibility of improving Duchenne muscular dystrophy by targeting the NLRP3 inflammasome. Although the double-edged sword effect of modulating the NLRP3 inflammasome in tumorigenesis is well documented, the role of the NLRP3 inflammasome in lymphomagenesis has not yet been explored. The latest article published in this Research Topic highlights that the NLRP3 inflammasome is activated in the diffuse large B-cell lymphoma microenvironment orchestrated by macrophages and discusses the potential therapeutic implications of the NLRP3 inflammasome in diffuse large B-cell lymphoma (Serna et al.).

Taken together, these interesting papers present new perspectives on the NLRP3 inflammasome and provide insight into exciting avenues of research that can enhance our understanding of the NLRP3 inflammasome for biomedical applications.

## Author contributions

K-FH wrote and finished the manuscript. K-FH is the guarantor of the article. All authors contributed to the article and approved the submitted version.
